# P03-013-B - Inaugural palate nodule of a systemic sarcoidosis

**DOI:** 10.1186/1546-0096-11-S1-A211

**Published:** 2013-11-08

**Authors:** M Mael-Ainin, M Bouaddi, K El Morabite, S Abil, A Abdou, K Senouci, B Hassam, I El Meknassi

**Affiliations:** 1 Dermatologie - Vénérologie, Centre Hospitalier Universitaire ibn Sina, Morocco; 2 Centre of Anatomic Pathology, United Nations, Rabat, Morocco

## Introduction

Oral manifestations of the sarcoidosis are rare. We report a case of a systematic sarcoidosis revealed by a nodule of the palate.

## Objectives

To emphasize on mucosa localization of sarcoidosis when addressing nodular lesion of the palate in order to allow an appropriate treatment.

## Methods

Mrs M. L a 29-year-old woman, presented an isolated nodule of the soft palate. The nasofibroscopy was normal, the histology showed a non specific inflammatory infiltrate. Two years later, the patient presented papulonodular lesions of the face associated with anosmia and a second stage dyspnea of NYHA.

## Results

Oral sarcoidosis is rare. Labial and lingual effects are at the forefront; effects of the palate remain exceptional. The lesions may take the form of a nodule, ulceration or perforation. Treatment based on systemic corticosteroids, sometimes with the addition of immunosuppressives or synthetic antimalarials.

## Conclusion

Sarcoidosis of the soft palate is rare and probably underdiagnosed. The risk of perforation or occurrence of systemic localization requires a careful examination of the oral mucosa in all patients with sarcoidosis.

## Competing interests

None Declared.

